# Image-Based Evaluation of Irradiation Effects in Brain Tissues by Measuring Absolute Electrical Conductivity Using MRI

**DOI:** 10.3390/cancers13215490

**Published:** 2021-10-31

**Authors:** Jin-Woong Kim, Ji-Ae Park, Nitish Katoch, Ji-ung Yang, Seungwoo Park, Bup-Kyung Choi, Sang-Gook Song, Tae-Hoon Kim, Hyung-Joong Kim

**Affiliations:** 1 Department of Radiology, Chosun University Hospital and Chosun University College of Medicine, Gwangju 61453, Korea; jw4249@gmail.com (J.-W.K.); sgsong71@gmail.com (S.-G.S.); 2 Division of Applied RI, Korea Institute of Radiological and Medical Science, Seoul 01812, Korea; jpark@kirams.re.kr (J.-A.P.); wjy11300@kirams.re.kr (J.-u.Y.); 3 Medical Science Research Institute, Kyung Hee University Hospital, Seoul 02447, Korea; nitish@khu.ac.kr (N.K.); josh_bk@naver.com (B.-K.C.); 4 Comprehensive Radiation Irradiation Center, Korea Institute of Radiological and Medical Science, Seoul 01812, Korea; swpark@kirams.re.kr; 5 Medical Convergence Research Center, Wonkwang University Hospital, Iksan 54538, Korea; tae_hoonkim@hanmail.net

**Keywords:** radiation therapy, electrical conductivity, ionizing radiation, tissue response, magnetic resonance imaging

## Abstract

**Simple Summary:**

Non-invasive quantification of radiation-induced damage is an important factor in radiation therapy to maximize radiation dose to cancer cells while minimizing damage to surrounding healthy tissue. Development of imaging biomarkers to assess post-RT effects accurately at an early stage is important for better prognosis and to individualize the management of brain tumors. Recent MR-based electrical conductivity imaging provides novel contrast information based on the concentration and mobility of ions constituting tissue and can exhibit high sensitivity in quantifying the therapeutic effect of ionizing radiation used in cancer treatment. This study suggests that the change in conductivity at different doses can provide a way to quantify the response of the tissue to irradiation, and the variation in conductivity with the elapsed time shows potential as a tool to monitor the therapeutic effect of radiation.

**Abstract:**

Radiation-induced injury is damage to normal tissues caused by unintentional exposure to ionizing radiation. Image-based evaluation of tissue damage by irradiation has an advantage for the early assessment of therapeutic effects by providing sensitive information on minute tissue responses in situ. Recent magnetic resonance (MR)-based electrical conductivity imaging has shown potential as an effective early imaging biomarker for treatment response and radiation-induced injury. However, to be a tool for evaluating therapeutic effects, validation of its reliability and sensitivity according to various irradiation conditions is required. We performed MR-based electrical conductivity imaging on designed phantoms to confirm the effect of ionizing radiation at different doses and on in vivo mouse brains to distinguish tissue response depending on different doses and the elapsed time after irradiation. To quantify the irradiation effects, we measured the absolute conductivity of brain tissues and calculated relative conductivity changes based on the value of pre-irradiation. The conductivity of the phantoms with the distilled water and saline solution increased linearly with the irradiation doses. The conductivity of in vivo mouse brains showed different time-course variations and residual contrast depending on the irradiation doses. Future studies will focus on validation at long-term time points, including early and late delayed response and evaluation of irradiation effects in various tissue types.

## 1. Introduction

Radiation therapy (RT) is a typical noninvasive method for cancer therapy that uses ionizing radiation delivered by a linear accelerator [1]. Ionizing radiation has sufficient energy to produce ions inside the human body at the molecular level, killing cancer cells by directly damaging the DNA of cancer cells or by creating charged particles that can damage the DNA within the cell [2,3,4,5]. The human central nervous system (CNS) is resistant to irradiation effects, but a higher irradiation dose can cause early and late delayed damages after RT [5,6,7]. In addition, some studies reported that the CNS is vulnerable to irradiation even at low doses [8,9,10]. Furthermore, several studies reported a decline in cognitive function after exposure to high and low irradiation doses [8,11,12,13].

An in vivo quantification of radiation-induced injury is a critical challenge in RT to maximize irradiation doses to cancer cells while minimizing damage to surrounding healthy tissue [14]. The difficulty in diagnosing radiation-induced injury is that it is complicated with a combination of total irradiation dose, dose per fraction, duration of irradiation, and its dependence on complex interactions between cell types [5,6,15]. In addition, RT-induced injury and response to treatment after RT appear slowly over several months [16]. Therefore, early identification of tissue damage by imaging methods has the advantages of predicting organ dysfunction, allowing re-correction of treatment strategies according to the individual patient’s condition and allowing interventions to minimize treatment-related injury before clinical symptoms appear [17,18]. To do this, the reliability and sensitivity evaluation of the applied imaging method is indispensable to enhance clinical implications in the early detection of tissue damage in RT.

Recently, Park et al. reported the potential of magnetic resonance (MR)-based electrical conductivity imaging as a sensitive tool to measure tissue response after irradiation [18]. The sensitivity of the conductivity was significantly higher than that of conventional MR imaging methods such as T2 and ADC mapping from in vivo animal imaging experiments [18]. Since the contrast mechanism of electrical conductivity originates from the concentration and mobility of ions that make up tissues [19,20,21], conductivity imaging has the advantage that it can provide direct information on ionizing radiation and tissue responses after irradiation. As a first feasibility study, they compared the sensitivity with different imaging methods at a single irradiation dose. Although it showed sufficient sensitivity to distinguish tissue responses, validation is required depending on various irradiation conditions, such as different doses and the time elapsed after irradiation. In particular, imaging of effects according to different doses and elapsed times is an important factor that can estimate the condition of tissues or organs with respect to reversible and irreversible responses by RT [6,13,16,17,18].

The purpose of this study is to show the experimental validation of MR-based electrical conductivity as an imaging biomarker to assess early tissue damage in RT. To verify the effect of ionizing radiation, phantom imaging using distilled water and saline solution, respectively, was performed to measure the changes in conductivity according to different irradiation doses. From in vivo mouse brain imaging, the absolute conductivity was measured to evaluate the irradiation effects through the changes at different doses and the time elapsed after irradiation. Finally, relative conductivity changes were calculated to quantify the extent of tissue response to irradiation.

## 2. Materials and Methods

### 2.1. Phantom Preparation for Validation

**Figure 1:**
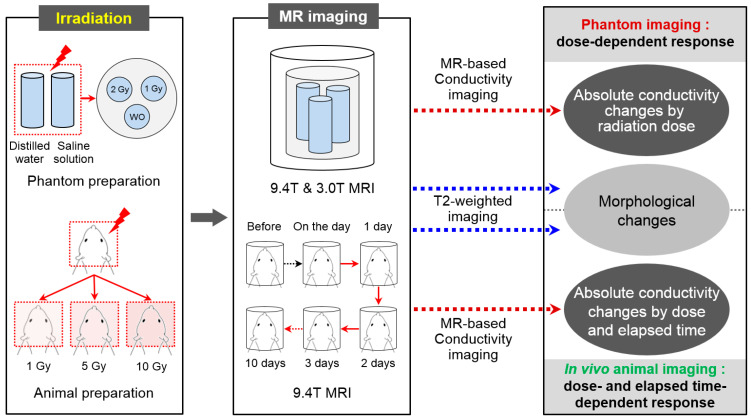
Schematic illustration of the experimental setup for MR-based electrical conductivity imaging to measure the irradiation effects in designed phantoms and in vivo mouse brains.

The phantom imaging experiment was first performed to verify the effects of ionizing radiation using electrical conductivity at different irradiation doses. Two types of phantoms were prepared using distilled water and saline solution, respectively. Three small cylindrical tanks containing distilled water and saline solution, respectively, consisting of without and with irradiation of 1 Gy and 2 Gy doses, were placed inside the cylindrical phantom (Figure 1). The background of the phantom was filled with agarose gel to support each tank. Phantom preparation for imaging experiments took about 30 min after irradiation.

### 2.2. Animal Preparation

A total of nine female 6-week-old BALB/c nude mice (weighing 20~23 g) were used for in vivo imaging experiments. Animal care, maintenance, and treatment in these studies were carried out according to the regulations of the Institutional Animal Care and Use Committee (IACUC, No. 2021-11) of the Korea Institute of Radiological & Medical Sciences (KIRAMS). The mice were divided into the following three groups: 1 Gy, 5 Gy, and 10 Gy irradiation dose (Figure 1). Each group consisted of three mice, and all mice were subjected to imaging experiments before and 0, 1, 2, 3, and 10 days after irradiation (Figure 1). To prevent their dribbling during the imaging experiments, we injected them with 0.1 mg/kg of atropine sulfate. Ten minutes later, we anesthetized each mouse with an intramuscular injection of 0.2 mL/kg of Zolazepam (Zoletil 50, Virbac, France).

### 2.3. Radiation Exposure

Neutron-beam irradiation was performed in two phantoms. The neutron-beam, including fast neutrons, was produced by irradiating a proton beam (20 μA, 35 MeV) on a beryllium target by using a KIRAMS MC-50 cyclotron (Scanditronix, Växjö, Sweden). The mean dose rate for fast neutrons was 94 mGy/min [22]. For in vivo brain imaging, mice were irradiated with a dose of 1 Gy, 5 Gy, and 10 Gy, respectively, using a small animal image-guided irradiation system X-RAD SmART (Precision X-ray Inc., North Branford, CT, USA) [23]. Before irradiation, micro-CT scanning was performed to ensure that the X-rays were correctly delivered to the mouse head.

### 2.4. Imaging Experiments

The phantom was placed inside the bore of an MRI scanner. T2-weighted imaging (T2WI) and electrical conductivity imaging were performed using a 9.4T MRI scanner (Agilent Technologies, Santa Clara, CA, USA) with a birdcage volume RF coil. For T2WI, a fast spin-echo multi-slice (FSE-MS) MR sequence was applied, and the imaging parameters were as follows: repetition time (TR), 3500 ms; echo time (TE), 30 ms; echo train length (ETL), 6; number of averaging, 2; slice thickness, 1 mm; number of slices, 5; matrix size, 128 × 128; field-of-view (FOV), 50 × 50 mm^2^; and total imaging time, 2 min 48 s.

For electrical conductivity imaging, a multi-echo multi-slice (MEMS) spin-echo MR sequence was applied to obtain the B1 map, which is used to recover high-frequency isotropic conductivity images in the magnetic resonance electrical properties (MREPT) method [18,24]. Before data acquisition, we applied a volume shimming method, with the volume defined to cover the imaging area. The imaging parameters were TR, 2200 ms; TE, 22 to 132 ms; number of echoes, 6; number of averaging, 5; slice thickness, 1 mm; number of slices, 5; matrix size, 128 × 128; FOV, 50 × 50 mm^2^; and total imaging time, approximately 23 min 46 s. 

Phantom imaging was also performed using a 3T MRI scanner (Magnetom Trio A Tim, Siemens Medical Solutions, Erlangen, Germany) to evaluate availability in the clinical MR system. The imaging parameters to acquire the T2WI and conductivity images were similar to those of the 9.4T MRI.

In vivo mouse brain imaging was performed before and after irradiation using a 9.4T MRI. The mouse was anesthetized with 2.5% isoflurane in oxygen and placed inside the MR scanner. T2WI and electrical conductivity imaging were acquired in the same way as described for phantom imaging.

### 2.5. Conductivity Measurement and Analysis

T2WI of the phantom and mouse brain was used to confirm the morphological changes after irradiation. MEMS images were reconstructed by a 2D fast Fourier transform using complex *k*-space data and then separated into magnitude and phase images to acquire electrical conductivity. The phase image was unwrapped using the PUMA algorithm [25], and the unwrapped phase images of each echo were averaged to achieve a higher SNR using a weighting factor. The final electrical conductivity was imaged from the optimized phase images. Details of conductivity reconstruction follow the work of Katoch et al. [21,24]. The conductivity of the phantom and mouse brains was measured to quantify the irradiation effects. Since electrical conductivity is a material property that provides an absolute value, we measured the conductivity values in the regions-of-interest (ROI) and calculated relative conductivity changes (%). The relative conductivity change (%), which indicates the sensitivity of conductivity on irradiation, was calculated following irradiation based on the values before irradiation.

## 3. Results

### 3.1. Phantom Imaging with Two Different Solutions

**Figure 2:**
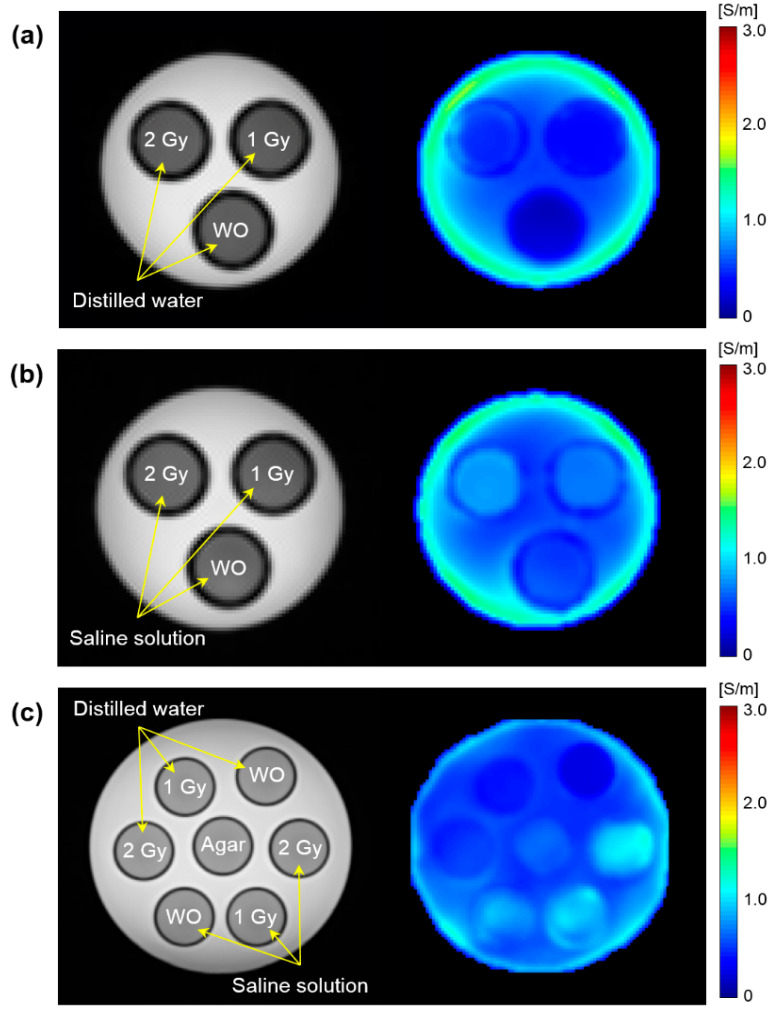
T2-weighted and electrical conductivity images of phantom with distilled water (**a**) and saline solution (**b**). The cylindrical tank was exposed to irradiation without and with 1 and 2 Gy doses and an imaging experiment was performed at 9.4T MRI. Phantom imaging with a combination of two solutions (**c**) was also performed on clinical 3T MRI. WO stands for without irradiation.

Figure 2 shows the T2WI and electrical conductivity images of the phantoms according to different irradiation doses. The phantoms using distilled water and saline solution were imaged separately in 9.4T MRI (Figure 2a,b), but combined in 3.0T MRI (Figure 2c). The morphological differences between the cylindrical tanks, which consisted of without and with irradiation of 1 Gy and 2 Gy dose, were not observed in the T2WI (first column). In contrast, the overall signal intensity and contrast in the conductivity image were clearly different depending on the irradiation doses (second column). 

**Figure 3:**
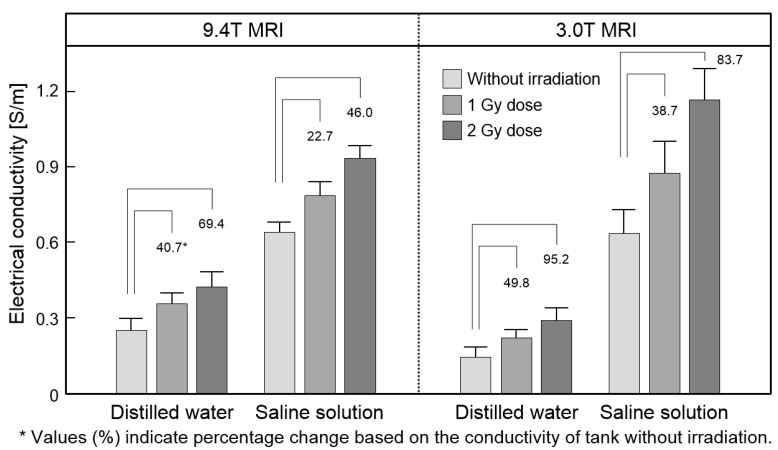
Analysis of phantom conductivity images obtained from 9.4T and 3T MRI. Absolute conductivity and relative conductivity change were obtained from the two solutions by different irradiation doses.

Figure 3 and Table 1 represent the analysis of absolute conductivity obtained from the phantom images at two different field strengths. Conductivity was measured in the ROI that covers each cylindrical tank. The conductivities of distilled water and saline solution were different according to the irradiation dose. The conductivity of the saline solution was higher than that of the distilled water in both strengths. The overall relative conductivity changes of the distilled water and saline solution were linearly changed by the irradiation dose at both field strengths. Specifically, the relative conductivity change in the distilled water was higher than that of the saline solution.

### 3.2. In Vivo Mouse Brain Imaging with Different Doses and Elapsed Times

**Figure 4:**
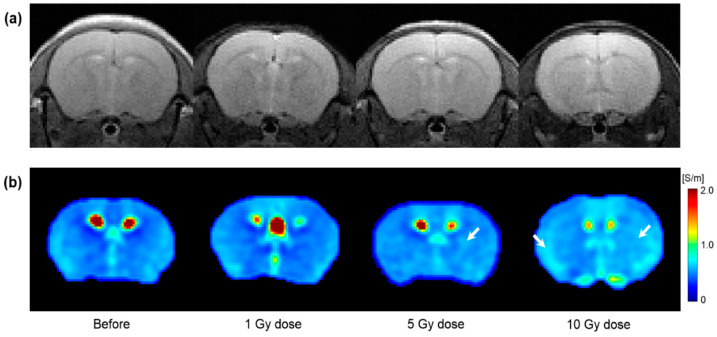
T2-weighted (**a**) and electrical conductivity (**b**) images of in vivo mouse brains with respect to tissue response by different irradiation doses. All images were acquired 1 day after irradiation. White arrows indicate the increase in conductivity contrast.

Figure 4 shows the T2WI and electrical conductivity image of in vivo mouse brains according to different irradiation doses. All images were acquired 1 day after irradiation. Compared to before irradiation, morphological differences were not observed in T2WI by the irradiation dose (Figure 4a). However, the conductivity images showed different contrasts by the irradiation dose (Figure 4b). Specifically, the conductivity image of the 10 Gy dose showed increased contrast throughout the brain region. The contrast was partially increased at 5 Gy. However, no clear contrast was observed in the 1 Gy dose.

**Figure 5:**
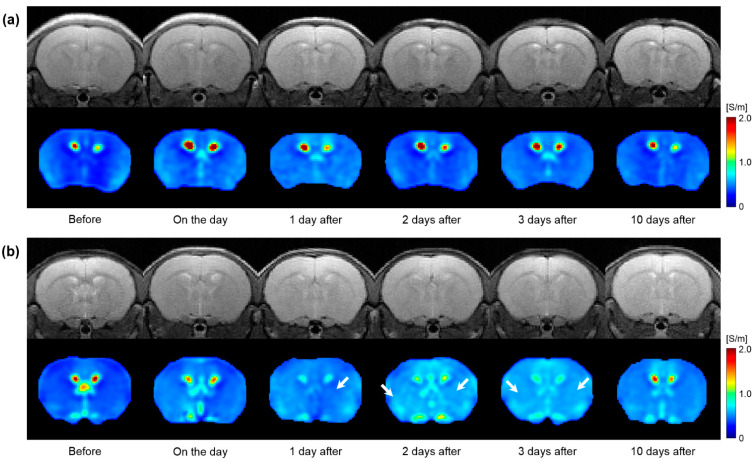
Time-course variations in T2-weighted and electrical conductivity images of in vivo mouse brains in response to 5 Gy (**a**) and 10 Gy (**b**) irradiation doses. White arrows indicate an increase in conductivity contrast.

Figure 5 shows a full time-course image of T2WI and electrical conductivity of the in vivo mouse brains after irradiation. All images were obtained at 5 Gy and 10 Gy irradiation doses (Figure 5a,b). Compared to before irradiation, the morphological changes were not clearly observed in T2WI (first row) at both irradiation doses. However, the conductivity images showed different contrast variations depending on the irradiation doses (second row). Specifically, the conductivity of the 5 Gy dose showed a slight increase up to 1 day afterwards and a decrease to 10 days. On the contrary, the conductivity of the 10 Gy dose showed an increase in contrast up to 2 days after and gradually decreased to 10 days. When comparing the conductivity at 10 days post-irradiation, the 5 Gy dose showed a similar contrast to the pre-irradiation, but the 10 Gy dose still had a residual contrast.

**Figure 6:**
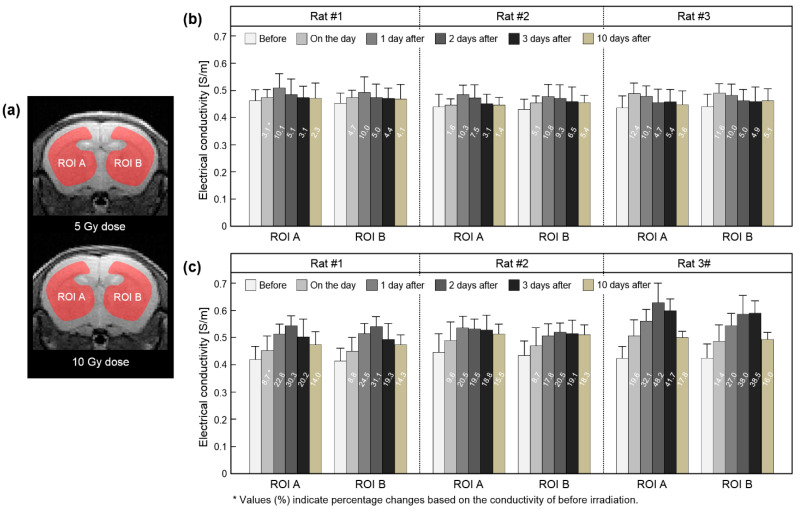
Measurement of absolute conductivity in brain tissues at full time points. ROIs were located in both hemispheres (**a**), and the conductivity was measured at 5 Gy (**b**) and 10 Gy (**c**) irradiation doses. The values inside the bar graph indicate relative conductivity changes.

Figure 6 shows the conductivity of in vivo brain tissues measured from a full time-course dataset at 5 Gy and 10 Gy irradiation doses. ROIs were placed to cover all brain tissues of both hemispheres, excluding cerebrospinal fluid (CSF) (Figure 6a). The conductivity of the 5 Gy dose increased slightly up to 1 day afterwards and decreased to 10 days (Figure 6b). The conductivity of the 10 Gy dose showed an increased contrast up to 2 days afterwards and gradually decreased to 10 days (Figure 6c). There was no clear difference between the ROIs at two irradiation doses. 

**Figure 7:**
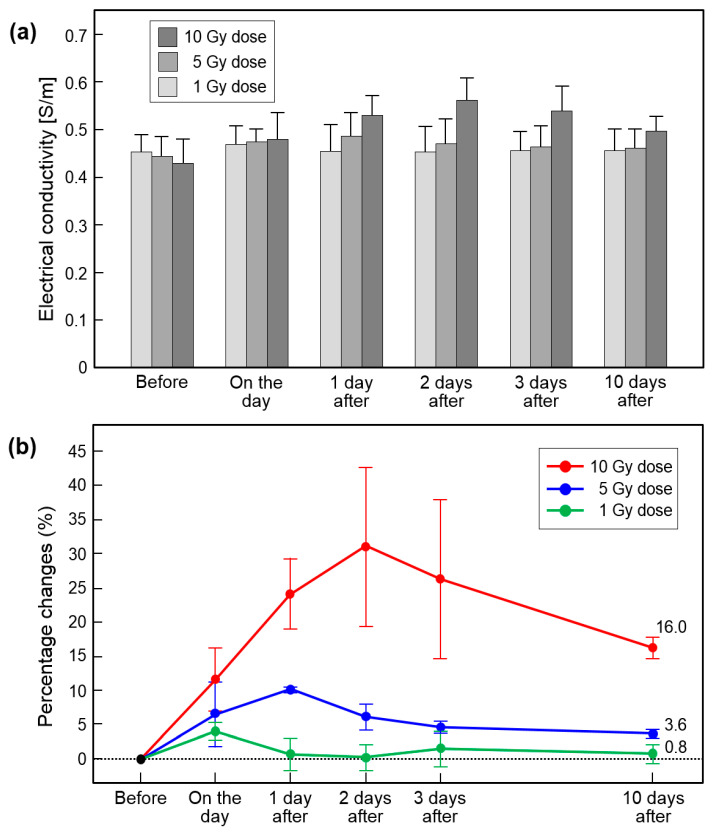
Analysis of in vivo brain conductivity images according to different doses and elapsed time after irradiation. Absolute conductivity (**a**) and relative conductivity changes (**b**) were acquired from doses of 1, 5, and 10 Gy with full time points.

Figure 7 and Table 2 show the measurement of absolute conductivity from in vivo mouse brains having a full time-course image by three irradiation doses. Conductivity was measured in all brain tissues except the CSF region. The conductivity change at the 10 Gy dose was the largest at all measurement times (Figure 7a). There was a slight change at the 5 Gy dose and almost no clear change at the 1 Gy dose (Figure 7a). The relative conductivity change represents the sensitivity of the irradiation effects on brain tissues based on the values found before irradiation (Figure 7b). The sensitivity of the 10 Gy dose increased by 30% up to 2 days after irradiation and then decreased. The 5 Gy dose increased by 10% up to 1 day after irradiation and then decreased. There was no clear change in the 1 Gy dose. When comparing the relative conductivity change 10 days after irradiation, there was a 16% difference in 10 Gy, 3.6% in 5 Gy, and 0.6% in the dose of 1 Gy (Figure 7b and Table 2).

## 4. Discussion

Recent MR-based electrical conductivity imaging reported higher sensitivity in tissue response to irradiation than T2 and ADC mapping [18]. However, few studies have reported the irradiation effects with conductivity changes under various irradiation conditions. In our phantom imaging, two phantoms were prepared with distilled water and saline solution to confirm the conductivity changes according to the different amounts of ionizing radiation. The phantom with distilled water is evidence that the conductivity changes stem from the ionization of water molecules into hydrogen and hydroxide ions. The phantom with physiological saline solution is a simple model assuming a body fluid composed of electrolytes with the isotonic concentration of the human body. From the results of phantom imaging, the conductivity of both phantoms increased linearly with increasing irradiation doses. The overall conductivity values of distilled water were clearly lower than those of the saline solution, but the relative conductivity changes were higher than those of the saline solution. 

The higher conductivity in the saline solution is related to differences in the amount and/or concentration of composing ions compared to those in distilled water. On the contrary, a high relative conductivity change in distilled water can be inferred from the differences in the types and/or numbers of composing ions responding to ionizing radiation. The absolute conductivity and relative conductivity changes differed slightly between the two MR field strengths, but the conductivity changes with increasing irradiation dose showed a similar pattern. This difference can be due to the frequency-dependent characteristic of electrical conductivity with the measurement frequency [26,27]. The linear change in conductivity by different doses in clinical 3T MRI showed potential for application in an in vivo human study.

An in vivo mouse brain imaging experiments was performed at three irradiation doses (1, 5, and 10 Gy) with an elapsed time of up to 10 days. One of the limitations of the previous MR-based conductivity imaging study of irradiation effects was the lack of information on tissue responses over the elapsed time and the need for evaluation by various irradiation conditions such as radiation source and dose [18]. In this study, the irradiation source for the phantom was a neutron-beam, and an X-ray was used for the in vivo mouse brain. It is well-known that the mechanism of causing ionizing radiation is different between the two sources, and that the relative biological effectiveness (RBE) of the neutron-beam is higher than that of the X-ray at the same dose [2,6,28,29,30,31]. The neutron-beam was used in the phantom to emphasize the effects of the ionizing radiation itself, while the X-ray was used in the mouse brain because it is predominantly used for cancer treatment [6,31]. 

From the in vivo mouse brain imaging results, the morphological differences were not clearly observed at different doses and elapsed times after irradiation. This can be inferred from the previous report that the changes in T2 relaxation time were within 10% despite the neutron-beam irradiation [18]. On the contrary, electrical conductivity clearly showed contrast changes with different doses and elapsed times after irradiation. In comparison, at a single time point after irradiation (Figure 4), the conductivity contrast was clearly distinguished at the 10 Gy dose from the other two lower doses. This indicates the reliability of electrical conductivity in that 10 Gy is an appropriate dose calculated considering the weight of the mouse [6]. In addition, the conductivity contrast of 10 Gy showed a different pattern with the elapsed time (Figure 5). Unlike the other two doses, the conductivity of 10 Gy showed a more sustained tissue response and residual conductivity even 10 days after irradiation. 

Our quantitative results in Figure 7 represent the sensitivity of MR-based electrical conductivity imaging. The relative conductivity change of the 10 Gy dose was highest at 31.3% 2 days after irradiation, the 5 Gy dose was at 10.2% 1 day after irradiation, and the 1 Gy dose was 4.0% on the day. This demonstrates two advantages derived from the measurement and quantification of electrical conductivity. First, conductivity changes can provide a dose-dependent response in irradiation together with the time point of maximum response. Second, the staging of tissue damage caused by irradiation can be possible by measuring absolute values. Therefore, conductivity contrast can be an imaging marker for the irradiation effects by the different irradiation conditions.

An interesting result of our study was the residual contrast in the 10 Gy dose 10 days after irradiation. The relative conductivity changes in 5 Gy and 1 Gy were less than 4%, but in 10 Gy was 16%. The irradiation effects can be reversible because cells exposed to a sublethal dose of irradiation can repair RT damage and cellular function can return to normal [6,32,33,34,35,36]. Therefore, tissue damage may not be observed after recovery from RT damage unless the tissue is exposed to high doses of ionizing radiation. The time points in this study correspond to an acute response that is generally considered reversible. Based on this, we can infer that the residual contrast in the 10 Gy dose may gradually decrease to be the same as before irradiation, but this should be clarified in future studies.

This study has several limitations. First, there is a lack of evidence for a correlation between the imaging results and histological data. The correlation can better differentiate between RT-induced tissue damage by providing detailed information about the threshold dose for tissue response to RT. Second, the conductivity changes in this study focused on the acute brain response. To determine reversible or irreversible tissue responses for clinical significance, conductivity imaging of early and late delayed brain responses should be performed. Third, a conductivity image-based evaluation should be performed on various tissues or organs. Tissue injury varies from one tissue to another depending on the cellular composition, proliferation rate, and the mechanisms of response to radiation. Finally, together with the imaging data from a large sample size, statistical analysis between conductivity changes and tissue response is required at more time points for clinical applications.

## 5. Conclusions

Tissue response by irradiation progresses slowly over time; sensitivity is one of the important factors in the image-based evaluation of irradiation effects. Ionizing radiation generally produces ions or charged particles within the cells; measuring absolute conductivity in tissues can provide highly sensitive information on the response following RT. This study focused on the validation of MR-based electrical conductivity imaging from the designed phantom and in vivo mouse brain by irradiation doses and the elapsed time of irradiation. A linear increase in conductivity with different doses in the phantom images can be evidence for ionizing radiation. From the in vivo mouse brain, the change in conductivity at different doses can provide a way to quantify the response of the tissue to irradiation, and the variation in conductivity with the elapsed time shows potential as a tool to monitor the therapeutic effect of radiation. Future studies will focus on conductivity imaging of tumor patients following sufficient validation, including long-term time points that cover early and late delayed response, as well as irradiation effects on various tissue types.

## Figures and Tables

**Table 1:** Summary of electrical conductivity and relative conductivity change in phantom imaging. The measured conductivity indicates absolute values by irradiation dose. The relative conductivity change indicates the extent of irradiation effects based on the values without irradiation.

Phantom Imaging		Electrical Conductivity (S/m)			Relative Conductivity Change (%)		
		Without	1 Gy	2 Gy	Without	1 Gy	2 Gy
9.4T MRI	Distilled water	0.252 ± 0.048	0.355 ± 0.045	0.427 ± 0.055	-	40.7	69.4
	Saline solution	0.643 ± 0.044	0.789 ± 0.052	0.939 ± 0.051	-	22.7	46.0
3.0T MRI	Distilled water	0.146 ± 0.040	0.218 ± 0.039	0.284 ± 0.061	-	49.8	95.2
	Saline solution	0.637 ± 0.100	0.883 ± 0.131	1.170 ± 0.161	-	38.7	83.7

**Table 2:** Summary of electrical conductivity and relative conductivity change (in parentheses) from in vivo mouse brain imaging. The measured conductivity indicates absolute values of the tissue response at three doses. The relative conductivity change indicates the extent of tissue response to irradiation based on the values of pre-irradiation.

In Vivo Brain Imaging		Electrical Conductivity (S/m) (Relative Conductivity Change, %)					
		Before	On the Day	1 Day after	2 Days after	3 Days after	10 Days after
1 Gy	Rat #1	0.436 ± 0.043	0.449 ± 0.032 (3.0)	0.451 ± 0.055 (3.4)	0.439 ± 0.046 (0.7)	0.449 ± 0.035 (3.2)	0.432 ± 0.042 (−0.9)
	Rat #2	0.463 ± 0.032	0.480 ± 0.033 (3.6)	0.456 ± 0.054 (−1.5)	0.453 ± 0.045 (−2.1)	0.477 ± 0.041 (3.0)	0.469 ± 0.058 (1.2)
	Rat #3	0.455 ± 0.041	0.479 ± 0.060 (5.4)	0.453 ± 0.071 (−0.4)	0.462 ± 0.079 (1.5)	0.447 ± 0.043 (−1.7)	0.464 ± 0.047 (2.0)
5 Gy	Rat #1	0.459 ± 0.038	0.477 ± 0.028 (3.9)	0.506 ± 0.053 (10.1)	0.482 ± 0.053 (5.0)	0.477 ± 0.039 (3.8)	0.474 ± 0.054 (3.2)
	Rat #2	0.436 ± 0.044	0.450 ± 0.026 (3.3)	0.482 ± 0.041 (10.6)	0.472 ± 0.050 (8.4)	0.457 ± 0.045 (4.8)	0.451 ± 0.029 (3.4)
	Rat #3	0.438 ± 0.047	0.490 ± 0.037 (12.0)	0.482 ± 0.040 (10.1)	0.459 ± 0.047 (4.9)	0.460 ± 0.049 (5.1)	0.457 ± 0.046 (4.3)
10 Gy	Rat #1	0.417 ± 0.048	0.454 ± 0.053 (8.8)	0.516 ± 0.038 (23.6)	0.545 ± 0.037 (30.7)	0.499 ± 0.063 (19.7)	0.476 ± 0.041 (14.1)
	Rat #2	0.439 ± 0.061	0.480 ± 0.069 (9.2)	0.524 ± 0.042 (19.2)	0.527 ± 0.035 (20.0)	0.523 ± 0.052 (19.0)	0.513 ± 0.036 (16.8)
	Rat #3	0.425 ± 0.049	0.497 ± 0.061 (17.0)	0.551 ± 0.047 (29.5)	0.608 ± 0.073 (43.1)	0.596 ± 0.046 (40.0)	0.497 ± 0.026 (16.9)

## Data Availability

The data presented in this study are available upon request from the corresponding author. The data are not publicly available for confidentiality reasons.
